# Does Personality Influence the Quality of Life of Patients with Brain Tumors Treated with Radiotherapy?

**DOI:** 10.3390/cancers17010131

**Published:** 2025-01-03

**Authors:** Agnieszka Pilarska, Anna Pieczyńska, Krystyna Adamska, Katarzyna Hojan

**Affiliations:** 1 Department of Rehabilitation, Greater Poland Cancer Centre, 61-866 Poznan, Poland; agnieszka.pilarska@wco.pl (A.P.); katarzyna.hojan@wco.pl (K.H.); 2 Department of Occupational Therapy, Poznan University of Medical Sciences, 60-781 Poznan, Poland; 3 Department of Radiotherapy, Greater Poland Cancer Centre, 61-866 Poznan, Poland; krystynaadamska@ump.edu.pl; 4 Department of Electroradiology, Poznan University of Medical Sciences, 61-701 Poznan, Poland

**Keywords:** brain tumor, radiation, well-being, cancer rehabilitation, psycho-oncology

## Abstract

**Background:** Understanding the role of personality traits in shaping treatment outcomes is crucial given the multifaceted challenges posed by brain tumors and the significant adverse impact of radiotherapy (RT) on patients’ well-being. **Purpose:** This study aimed to provide insights into how personality traits affect psychosocial well-being and quality of life during RT in patients with high-grade malignant brain tumors. **Methods:** Personality traits in patients with high-grade glioma were assessed using the Eysenck Personality Questionnaire-Revised (EPQ-R). Quality of life was analyzed using EORTC questionnaires: the Questionnaire-Core 30 (QLQ-C30) and the Brain Cancer Module (QLQ-BN20). Patients were evaluated before RT, immediately after 6 weeks of RT, and 3 months post-RT. **Results:** Neuroticism predicted emotional function only three months post-RT. Extraversion decreased quality of life in global health status (third assessment), role function (second assessment), and emotional function (second and third assessments) but improved cognitive (first assessment) and social function (second assessment). The trait associated with lying was linked to a better quality of life in all domains except physical and cognitive function. Anxiety predicted a lower quality of life in brain tumor patients across all domains at various stages of RT treatment. **Conclusions:** This study advances our understanding of the psychosocial aspects of brain tumor care by highlighting the influence of personality traits on quality-of-life outcomes during RT. Identifying high-grade glioma patients at greater risk of a diminished quality of life based on personality profiles allows healthcare professionals to tailor interventions to address specific psychosocial needs, ultimately enhancing patient outcomes and holistic care during oncological treatment.

## Simple Summary

This study explores how personality traits influence the quality of life (QoL) in patients with high-grade malignant brain tumors undergoing radiotherapy (RT). By assessing traits such as neuroticism, extraversion, and anxiety, alongside QoL indicators, this research identifies how psychological profiles shape emotional, cognitive, and social outcomes during and after treatment. Understanding these relationships can help healthcare professionals personalize supportive care strategies, ultimately improving patient well-being and treatment experiences. The findings emphasize the need for integrating psychological assessments into routine oncology care, offering practical guidance to enhance holistic cancer management and potentially influencing future research on psycho-oncological interventions.

## 1. Introduction

Brain tumors represent a significant health burden worldwide, with substantial implications for patients’ quality of life (QoL) and overall well-being [1]. The diagnosis and management of brain tumors pose complex challenges due to the heterogeneity of tumor types, variability in treatment responses, and potential neurocognitive sequelae [2,3]. Among the various treatment modalities, radiotherapy (RT) remains a cornerstone in the therapeutic armamentarium for high-grade brain tumors, aiming to achieve tumor control and alleviate symptoms.

Despite advancements in radiation therapy techniques, the impact of treatment on patients’ QoL remains a critical consideration in clinical practice and research [4]. While clinical outcomes such as survival and disease control are traditionally prioritized in oncology research, there is increasing recognition of the importance of psychosocial factors in shaping patient experiences and treatment outcomes. Personality traits have emerged as significant determinants of psychological adjustment, coping strategies, and QoL in cancer patients [5,6,7,8,9,10,11].

Understanding the influence of personality on treatment-related outcomes is crucial for delivering patient-centered care and optimizing supportive interventions tailored to individual needs [12]. Previous studies have demonstrated associations between specific personality traits and psychological distress, coping mechanisms, and health-related QoL outcomes in cancer patients [13,14,15,16,17]. Interestingly, Gempt et al. [18], in their research, did not find any significant factors (tumor entity, location) influencing the personality traits of patients with neuroepithelial tumors. They did note that neuroticism was associated with depression, while extraversion exhibited an opposite correlation. In patients with brain tumors, the relationship between personality traits and emotional and cognitive health, as well as prognosis, was examined. Emotional stability and openness were associated with a lower severity of depressive and anxiety symptoms, while extraversion was linked to better cognitive functioning, independent of demographic and clinical risk factors. Openness predicted a lower risk of mortality in patients with benign brain tumors [19]. However, limited research has focused specifically on the impact of personality traits on QoL outcomes in brain tumor patients undergoing radiation treatment.

Therefore the objective of present study was to fill this gap by investigating the influence of personality factors, specifically neuroticism, psychoticism, and extraversion, on the QoL experienced by patients undergoing radiation treatment for high-grade brain tumors (glioma). Using validated measures to assess personality traits and quality of life, we aimed to elucidate the interplay between personality and well-being in this cancer patient population. Through a comprehensive analysis, we sought to contribute valuable insights to the field of neuro-oncology and present evidence-based approaches to support the provision of holistic care for brain tumor patients undergoing RT.

## 2. Materials and Methods

### 2.1. Characteristic of Study

This clinical study was designed as a prospective investigation and conducted between September 2021 and December 2023 at the Radiotherapy Department of the Greater Poland Oncology Center in Poznań. The research was funded by the National Science Centre, Poland (Project No. 2020/37/B/NZ7/01122) and registered in the ClinicalTrials.gov database (ID No. NCT05192447, accessed on 9 September 2023). The study protocol received approval from the Ethics Committee of the Poznań University of Medical Sciences (approval No. 505/21). Written informed consent was obtained from all participants prior to their inclusion in the study.

### 2.2. Study Scheme

Participants provided demographic information, including age, gender, and medical history. Personality and trait anxiety were assessed once, before the initiation of radiotherapy. Quality of life and state anxiety levels were evaluated three times: (I) one day before RT, (II) one day after 6 weeks of RT, and (III) three months later.

### 2.3. Participants

We enrolled participants for this study according to the study criteria. Eligibility criteria included a patient with a confirmed diagnosis of stage III or IV glioma according the WHO classification [20] and selected by oncologists for intensity-modulated radiotherapy (IMRT). Participants were approached during their scheduled appointments for radiotherapy sessions at the dedicated medical facility using a conventional fractionation of 2 Gy per dose to the total dose of 60 Gy [21]. The study included patients in good general condition (according to the Eastern Cooperative Oncology Group (ECOG), between 0 and 1). Patients with multiple cancers or metastatic cancers or with psychiatric diseases, as well as with other neurological diseases (e.g., multiple sclerosis, Parkinson’s disease, after stroke, meningitis, etc.) or significant clinical circulatory failure (≥III NYHA) were excluded.

### 2.4. Personality Trait Assessment

Personality was assessed to evaluate neuroticism, psychoticism, and extraversion using the Eysenck EPQ-R Personality Questionnaire in its shortened version [22]. These authors developed an abbreviated version (EPQ-R (S)) to enable the study to take place in situations where, for various reasons, the time allocated to the study was limited. The questionnaire is applicable to people aged 16–69. EPQ-R (S) contains 48 questions, to which the respondent answers “yes” or “no”. There is also a table on the sheet, where the examiner enters the results in 4 scales (psychoticism, extraversion, neuroticism, and lie), and the corresponding score. For an answer compliant with the key, the respondent receives 1 point, for an answer not compliant with the key—0 points. The result in a given scale is the sum of points obtained for answers to the questions included in it. Each scale consists of 12 items.

### 2.5. Anxiety-Level Measures

The State–Trait Anxiety Inventory (STAI), developed by Spielberger et al. [23], was utilized to assess anxiety levels in participants. This instrument is specifically designed to evaluate two distinct aspects of anxiety: anxiety as a transient and situational state (state anxiety) and anxiety as a relatively stable personality trait (trait anxiety). The STAI comprises two subscales—X-1 for state anxiety and X-2 for trait anxiety—each containing 20 items. Respondents answer each item using a four-point Likert scale, ranging from 1 (“definitely no”) to 4 (“definitely yes”). The anxiety level is calculated by summing the scores for all items, with values ranging from 1 to 10. Higher scores indicate a greater level of anxiety.

Spielberger defines trait anxiety as an acquired behavioral tendency that predisposes individuals to perceive a broad range of situations as threatening, even when they are objectively harmless. This predisposition leads to anxiety responses that are disproportionate to the actual level of threat. The definition highlights the learned nature of this emotional response, distinguishing it from situational anxiety.

### 2.6. Quality of Life Assessment

Quality of life was assessed using the EORTC Core Questionnaire (QLQ-C30), developed by the European Organization for Research and Treatment of Cancer. The QLQ-C30 evaluates various quality of life domains, including physical, emotional, and social functioning, as well as symptomatology [24]. It consists of 30 items, with responses scored on a Likert scale from 1 (“not at all”) to 4 (“very much”). Higher scores indicate a better quality of life in functional domains, whereas higher scores on symptom-related items reflect a greater symptom burden.

To complement the QLQ-C30, the Brain Cancer Module (QLQ-BN20) was also utilized. This supplementary questionnaire includes four multi-item scales that address uncertainty about the future, visual disturbances, motor dysfunction, and communication difficulties. Additionally, it contains seven single-item measures assessing specific symptoms such as headaches, seizures, drowsiness, hair loss, itchy skin, leg weakness, and bladder control. Scoring for the QLQ-BN20 follows the same principles as the symptom scales and single items in the QLQ-C30 [25]. The use of these questionnaires was approved by the EORTC for this study

### 2.7. Statistical Analysis

The statistical analysis was conducted using Statistica 13.3 software (TIBCO Software, Krakow, Poland). To determine the sample size, a power analysis was performed using G*Power [26], assuming Cohen’s f for the repeated measures ANOVA at 0.25, an alpha level of 0.05, power of 0.95, and three time points. These parameters yielded a minimum required sample size of *N* = 31, ensuring adequate statistical power to detect significant effects. 

The choice of statistical models was guided by the nature of the data and the study objectives. The Shapiro–Wilk test was used to assess the normality of distributions. Differences over time were examined using repeated measures ANOVA, as this method is suitable for analyzing changes within individuals across multiple time points. Mauchley’s test was conducted to verify the assumption of sphericity; if this assumption was violated, the Greenhouse–Geisser correction was applied to adjust for deviations. Tukey’s post hoc test was used to identify specific differences between time points, providing a robust framework for pairwise comparisons while controlling for Type I errors.

For variables with non-normal distributions or ordinal data, Friedman’s test was employed as a non-parametric alternative to repeated measures ANOVA. Dunn’s post hoc test was subsequently applied for pairwise comparisons, offering a methodologically sound approach to analyzing these data.

Spearman’s correlation analysis was used to explore relationships between personality traits and specific domains of quality of life, as this non-parametric method is appropriate for ranked data and non-linear relationships. Additionally, multiple regression analyses were conducted to identify factors influencing QoL ratings at various stages of radiotherapy treatment. This model was chosen to account for the simultaneous effects of multiple predictors, providing a comprehensive understanding of the key variables impacting quality of life.

## 3. Results

### 3.1. Participant Characteristics

**Figure 1:**
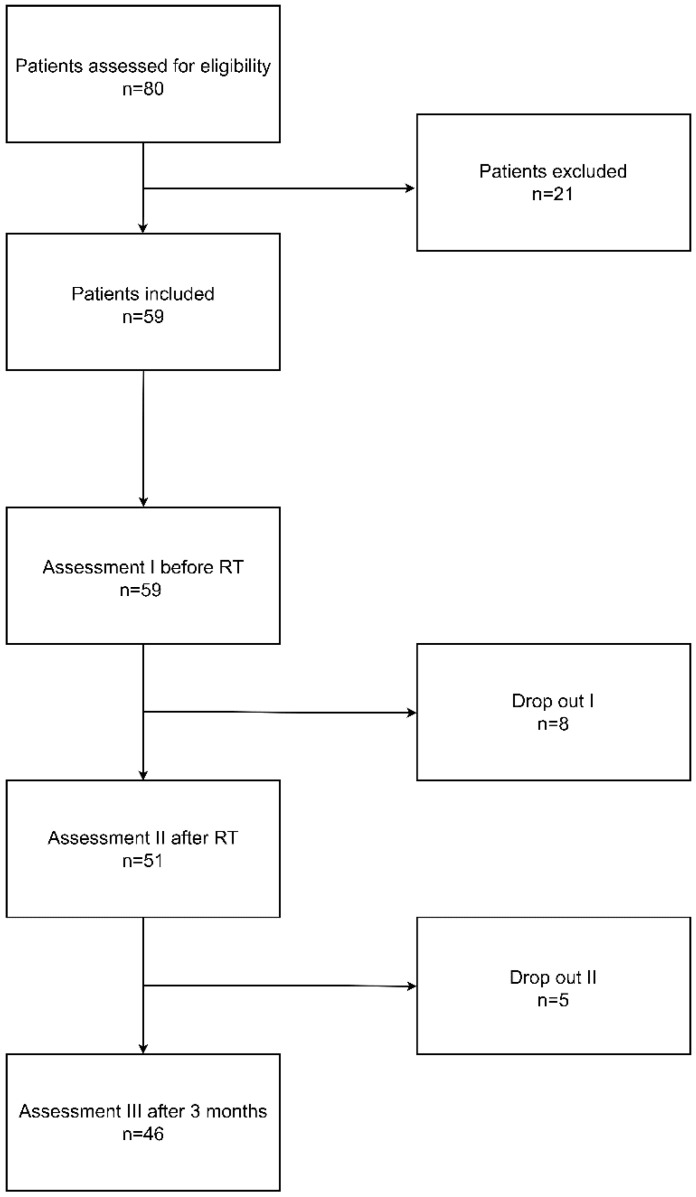
Study flow diagram.

A total of 80 patients were initially screened for eligibility in the study. Among these, 21 individuals were excluded for the following reasons: lack of consent (*n* = 10), age over 70 years (*n* = 2), presence of more than two tumors (*n* = 5), neurological disorders (*n* = 2), and NYHA class III or IV heart failure (*n* = 2). During the first assessment (I), eight participants withdrew their consent. At the second assessment (II), two patients experienced disease recurrence, one patient passed away, and two additional participants withdrew from the study. The study flow diagram is presented in Figure 1.

Of the 46 patients who underwent the final assessment, the majority were male. Most of the participants had undergone complete tumor resections and were receiving chemotherapy during the study period. Detailed patient characteristics are presented in Table 1.

### 3.2. Questionnaire Outcomes

#### 3.2.1. Personality Traits

**Figure 2:**
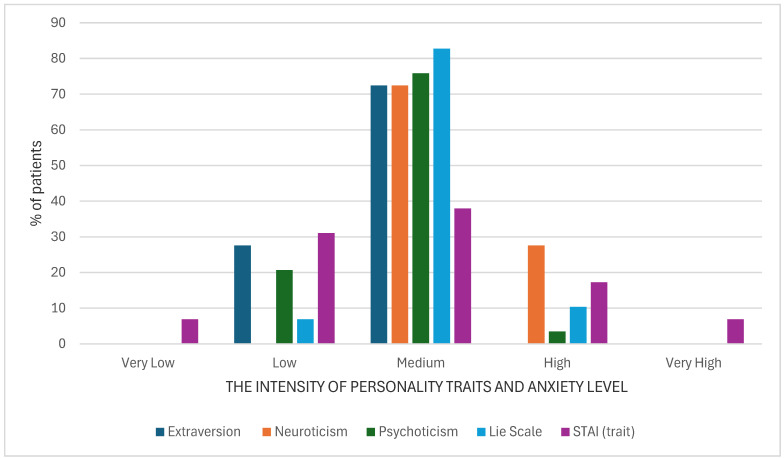
Percentage of patients presenting a severity of particular personality traits and anxiety level.

An analysis of the Eysenck Personality Questionnaire-Revised (EPQ-R) revealed varying levels of personality traits among the participants (*n* = 46). The greatest number of respondents achieved a moderate intensity of the studied variables; high scores were most often related to the traits of neuroticism, low extraversion, and psychoticism. It is worth noting that among the respondents, no one obtained a low result in terms of neuroticism (Figure 2).

#### 3.2.2. Anxiety Level

**Figure 3:**
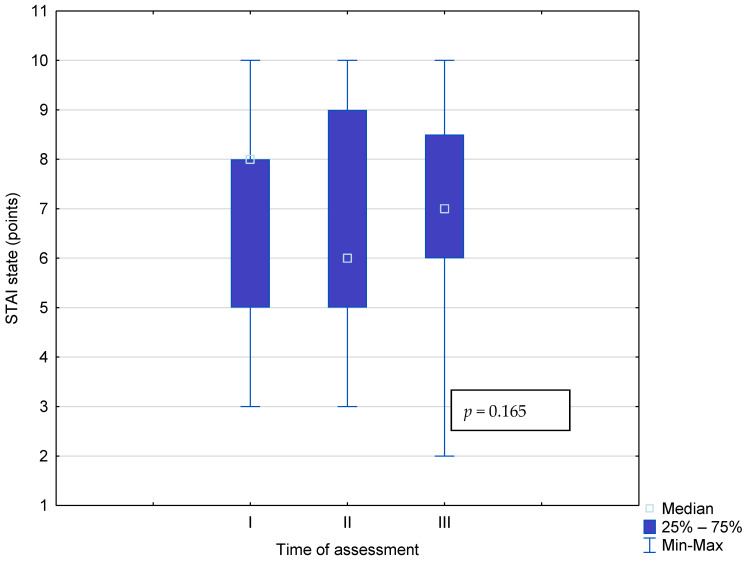
Change in the level of anxiety as a state at different stages of the study.

The level of anxiety among study participants (*n* = 46), measured as a state at a given moment, remained at a similar moderate level throughout the study. No statistical differences were observed in the median scores during the first, second, and third measurements, *p* = 0.165. (Figure 3.)

#### 3.2.3. Quality of Life

The assessment of QoL using the QLQ C-30 in functional domains showed statistically significant differences between measurements over time for all domains. The highest scores, indicating the best QoL in the domains of global health status, physical functioning, emotional functioning, and cognitive functioning, were observed before the commencement of RT. For social functioning, the mean scores before RT were similar to the mean scores three months after the completion of radiation treatment. The role functioning domain received the highest scores in the third assessment.

In the symptom domains, statistically significant differences between measurements were found only in the assessments of constipation and financial difficulties (with the worst score three months after the completion of RT).

In the assessment of QoL related to specific symptoms for patients with brain tumors using the QLQ BN20, differences over time in the evaluations of individual domains were found only in one domain—leg weakness, which received the worst evaluation three months after RT. Detailed results of quality of life in the individual domains, along with their changes over time, are presented in Table 2.

### 3.3. Correlational Analysis

Table 3 presents correlations of the intensity of personality traits and anxiety as a trait with individual domains of quality of life at three time points of the study.

### 3.4. Linear Regression

Analyzing multivariate regression models revealed that personality traits explained only certain areas of patients’ quality of life. Neuroticism was a predictor of emotional function only three months after the completion of RT. Extraversion was a significant factor that decreased QoL in the domains of global health status (third assessment), role function (second assessment), and emotional function (second and third assessments). However, extraversion had a positive impact on cognitive function (first assessment) and social function (second assessment). Psychoticism was a significant predictor of low QoL ratings in the domains of global health status and emotional function three months post-RT. The personality trait associated with lying was linked to better QoL ratings in all domains except physical and cognitive function.

Anxiety as a trait was a significant predictor of lower QoL ratings across all domains at various stages of RT treatment. Detailed results of the regression analysis are presented in Table 4.

## 4. Discussion

The present study investigated the impact of personality traits on QoL among high-grade brain tumor patients undergoing radiotherapy (IMRT). The findings underscore the complex interplay between personality factors, psychological well-being, and treatment-related outcomes, providing critical insights into patient-centered care in neuro-oncology. Cancer should be understood as a multifaceted and intricate process, where psychosocial factors play a crucial role in both its development and progression. Conversely, the disease itself imposes substantial psychosocial challenges and triggers significant changes in patients’ lives [27]. 

While it is well documented that health behaviors and socioeconomic status significantly impact cancer development and prognosis, the role of stress and personality factors is less clear [28].

The analysis revealed that neuroticism, a personality trait characterized by emotional instability and anxiety, was consistently associated with poorer QoL outcomes across various domains, both before and after RT. This is consistent with previous research indicating that higher neuroticism levels are linked to increased psychological distress and lower QoL [16] and lower satisfaction of life in cancer patients [29,30]. Specifically, in our study, neuroticism was negatively correlated with global health status, emotional functioning, and social functioning, while also being associated with a greater severity of symptoms such as nausea, vomiting, and loss of appetite. Previous studies have indicated that both neuroticism and introversion (the opposite of extraversion) are associated with a higher risk of emotional distress in cancer patients, which in turn correlates with lower levels of physical and mental health, as well as overall well-being [31,32]. Other authors, in a study of a population with prostate cancer, observed that men with high levels of neuroticism prior to treatment reported significantly higher rates of overall urinary problems and sexual dysfunction [33]. With the growing focus on “personalized medicine”, which involves adapting healthcare interventions to the specific traits of each patient, including personality factors, neuroticism could be particularly important. People with higher levels of neuroticism may find it challenging to manage negative emotions, often turning to unhealthy coping mechanisms (such as poor dietary choices or avoiding exercise). As a result, this may contribute to poorer health outcomes and a diminished quality of life [34].

In studies by Bunevicius et al. [19], it was demonstrated that extraversion was associated with better cognitive functioning, independent of demographic and clinical risk factors, in patients with brain tumors. Similarly, in our study, multivariate analysis revealed a positive effect of extraversion on the social and cognitive domains of QoL. These results are consistent with the typical traits of extraverted individuals, who are generally self-assured and outgoing. Extraverts tend to have a more positive perspective on their experiences and maintain an optimistic viewpoint, which enhances their ratings of life satisfaction. Individuals with high levels of extraversion often exhibit increased physical activity, improved sleep quality, and a reduced risk of depression and anxiety [30]. Consequently, they typically report greater life satisfaction. All of these elements may contribute to a protective effect on the QoL during radiation treatment for patients with brain tumors. Nevertheless, extraversion may also have nuanced impacts, as extraverted patients may face challenges in adjusting to treatment-related physical limitations, potentially resulting in temporary declines in QoL during specific stages of therapy. These challenges could include a sense of frustration or reduced self-efficacy when physical limitations interfere with their typically active and outgoing lifestyle. For example, extraverted individuals might find it particularly difficult to cope with the prolonged fatigue or social isolation that can accompany certain stages of radiotherapy. Understanding these potential vulnerabilities is critical for developing comprehensive care strategies. Future research should further explore these dynamics to identify tailored interventions that amplify the protective aspects of extraversion, such as encouraging alternative forms of social interaction or physical activity that accommodate patients’ treatment-related constraints.

Psychoticism showed minimal correlations with QoL measures. Earlier data indicate that, one year after completing treatment, cancer patients tend to exhibit higher levels of neuroticism and psychoticism compared to the general population, likely as a result of changes in their work situation and elevated distress levels [35]. Interestingly, the trait of lying, typically associated with social desirability and response bias, exhibited positive correlations with QoL outcomes three months post-treatment, suggesting that patients with higher scores in this trait reported a better QoL. This finding could reflect a coping mechanism where patients present themselves in a more favorable light to manage the psychological burden of their condition [36]. It could also suggest that the ability to maintain a socially desirable self-presentation helps mitigate some of the stress and anxiety associated with their illness. Although psychoticism itself did not significantly affect QoL during treatment, these findings highlight the importance of nuanced psychological assessments that consider personality traits and coping styles as interconnected factors influencing patient outcomes. This underlines the need for a broader, integrative approach in clinical practice, where psychological and behavioral profiles are regularly assessed alongside clinical parameters. For example, patients exhibiting traits associated with psychoticism may benefit from targeted psychosocial interventions aimed at enhancing emotional regulation and stress management, even if the immediate impact on QoL appears minimal. Moreover, the interplay between psychoticism and coping mechanisms such as denial or avoidance should be explored further, as these could mediate long-term outcomes post-treatment. By incorporating regular psychological evaluations into routine care, healthcare providers can better understand how even subtle personality dynamics may influence patient adherence, resilience, and overall recovery trajectories. Future studies should also examine whether specific combinations of traits, such as high psychoticism paired with neuroticism, create compounding vulnerabilities or unique opportunities for intervention, thereby improving personalized care strategies.

The stability of anxiety levels measured throughout the study indicates that while RT significantly impacts physical and functional domains of QoL, the psychological burden remains consistently moderate. This observation is critical as it highlights the need for continuous psychological support throughout the cancer treatment trajectory for patients with brain tumor. Prior studies have shown that sustained psychological interventions can mitigate anxiety and improve overall well-being in cancer patients [28,37]. Tailored interventions, such as cognitive behavioral therapy (CBT) and mindfulness-based stress reduction (MBSR), have been particularly effective in addressing the unique psychological needs of neuro-oncology patients [38,39,40,41]. CBT, for example, can help patients with high neuroticism or anxiety develop healthier coping mechanisms, reframe negative thoughts, and improve emotional regulation. Similarly, mindfulness techniques, including meditation and stress reduction exercises, have been associated with reductions in anxiety, improvements in emotional well-being, and enhanced QoL. These interventions could be integrated into routine care for neuro-oncology patients to provide tailored psychological support and optimize treatment outcomes.

This study is not without certain limitations. Firstly, personality traits were assessed only before the start of radiotherapy, which may not fully reflect their changes during treatment. As shown in the literature [42,43,44], brain tumors, especially those located in the frontal lobe, can influence changes in patients’ personality, which may be further exacerbated by the treatment process, including radiotherapy neurotoxicity. Therefore, the results regarding the impact of personality traits on quality of life should be interpreted with caution, as they may not account for potential dynamic personality changes during therapy. Future studies should include an assessment of personality traits at different time points, both during and after radiotherapy, to better understand their dynamics and associations with patients’ quality of life.

This study was conducted in a single center in Poland, which may limit the generalizability of the results to other populations and cultural contexts. Interactions between personality traits and quality of life may be modified by cultural, social, and economic factors. The Polish population may differ from populations in other countries, for example in terms of the healthcare system, approach to psychological support, or perception of quality of life. Multicenter and international studies are needed to confirm our results, which will allow us to assess their universality in different clinical and cultural settings.

Additionally, this study did not take into account other factors that could impact patients’ quality of life, such as social support. Nevertheless, it is important to highlight that this is the first study to analyze the relationship between personality traits in primary brain tumor patients undergoing RT, including a three-month follow-up period.

## 5. Conclusions

To summarize, our study highlights the significant influence of personality traits, particularly neuroticism, on the QoL of high-grade brain tumor patients undergoing radiation treatment. These insights advocate for incorporating personality assessments into clinical practice to identify patients at risk of poor psychological adjustment and tailor interventions accordingly. Based on the results of the study, key guidelines for clinical practitioners can be identified. A high level of neuroticism emerged as a significant predictor of a reduced quality of life in patients, particularly in the areas of global health status, emotional functioning, and social functioning. While extraversion positively influenced cognitive and social functioning, it was also associated with deterioration in other QoL domains during specific stages of therapy. Conversely, a high level of anxiety as a trait was a notable risk factor for a lower QoL across all domains, both during and after radiotherapy. To efficiently identify patients requiring targeted support, the use of abbreviated screening tools is recommended, such as the EPQ-R (S) Personality Questionnaire, which assesses levels of neuroticism, extraversion, and psychoticism, and the STAI Anxiety Inventory, which provides a comprehensive evaluation of both state and trait anxiety.

Psychological support for patients with brain tumors should include interventions like cognitive behavioral therapy (CBT), which can significantly improve QoL, particularly for individuals with high levels of neuroticism. Future studies should also consider including caregivers, given their critical role in providing emotional support and coordinating care. Investigating the impact of caregivers’ personality traits on patient QoL, as well as assessing caregiver stress and burden, could inform the development of effective support strategies. The integration of therapeutic programs designed for both patients and their caregivers holds the potential to enhance overall care in neuro-oncology. Future research should explore targeted individual and group therapies that address the specific needs of patients with high neuroticism to enhance their QoL during and after RT. Additionally, longitudinal studies examining the long-term effects of personality traits on survivorship and post-treatment QoL are warranted to better understand and support this vulnerable population.

## Figures and Tables

**Table 1:** Demographic and clinical characteristics of participants.

Characteristic	Participants (*n* = 46) *n* (%) or Mean ± SD
Age, years	51.5 ± 13.2
Sex	
Female	20 (43.48)
Male	26 (56.52)
Stage of tumor according WHO 2021	
III	12 (26.09)
IV	34 (73.91)
Tumor location	
Right	20 (43.48)
Left	23 (50.00)
Both/other	3 (6.52)
Education	
Vocational	10 (21.74)
Technical	9 (19.57)
Secondary	15 (32.61)
High	12 (26.09)
Total resection	
Yes	29 (63.04)
No	17 (36.96)
Chemotherapy	
Yes	35 (76.09)
No	11 (23.91)

**Table 2:** Differences in patients’ assessment of quality of life at three assessment time points.

EORTC QLQ (Mean ± SD)	Before RT (*n* = 46)	After RT (*n* = 46)	After 3 Months (*n* = 46)	*p*-Value
QLQ-C30 functional domains ^1^				
Global health status	50.64 ± 23.83	42.98 ± 22.89	41.95 ± 21.88	<0.001 ^a,b^
Physical functioning	82.91 ± 16.24	76.84 ± 22.34	76.32 ± 21.13	0.049
Role functioning	81.62 ± 19.04	55.26 ± 30.54	93.10 ± 13.74	<0.001 ^a,c^
Emotional functioning	61.97 ± 24.17	49.56 ± 24.50	46.26 ± 26.97	0.003 ^a,b^
Cognitive functioning	78.63 ± 19.85	68.86 ± 21.63	67.82 ± 19.89	0.006 ^b^
Social functioning	50.86 ± 26.60	39.04 ± 28.28	50.57 ± 27.63	0.004 ^a^
QLQ-C30 symptom domains ^1^				
Fatigue	31.05 ± 20.74	40.94 ± 27.23	36.02 ± 17.98	0.087
Nausea and vomiting	2.99 ± 8.44	3.95 ± 9.03	6.90 ± 13.74	0.321
Pain	14.96 ± 17.85	18.42 ± 23.18	18.39 ± 20.09	0.854
Dyspnea	4.27 ± 11.29	6.14 ± 18.75	6.90 ± 20.66	0.651
Insomnia	29.06 ± 28.80	34.21 ± 28.46	35.63 ± 23.45	0.906
Appetite loss	6.84 ± 15.63	10.53 ± 17.51	16.09 ± 24.59	0.124
Constipation	18.80 ± 29.41	8.77 ± 16.77	9.20 ± 19.71	<0.001
Diarrhea	0.85 ± 5.34	1.75 ± 7.54	1.15 ± 6.19	0.607
Financial difficulties	18.80 ± 23.93	19.30 ± 24.05	22.99 ± 23.74	0.018
BN20 symptom domains ^2^				
Future uncertainty	33.82 ± 27.67	65.68 ± 40.42	33.85 ± 22.87	0.132
Headaches	14.71 ± 23.49	10.53 ± 15.92	8.33 ± 14.91	0.325
Visual disorders	16.34 ± 26.55	9.94 ± 17.33	16.67 ± 19.46	0.891
Seizures	11.76 ± 27.07	17.54 ± 32.14	12.50 ± 23.96	0.819
Motor dysfunction	19.28 ± 26.70	16.37 ± 22.03	15.97 ± 23.99	0.809
Communication deficit	11.11 ± 19.53	9.36 ± 12.43	15.28 ± 23.61	0.223
Drowsiness	33.33 ± 25.95	35.09 ± 23.50	33.33 ± 32.20	0.267
Hair loss	8.82 ± 17.03	12.28 ± 22.80	16.67 ± 21.08	0.883
Itchy skin	4.90 ± 11.98	7.02 ± 13.96	16.67 ± 32.20	0.867
Weakness of legs	12.75 ± 21.73	10.53 ± 22.37	27.08 ± 32.70	0.007
Bladder control	4.90 ± 11.98	3.51 ± 10.51	8.33 ± 25.82	0.368

^a^ Difference in a post hoc test between baseline and after RT. ^b^ Difference in a post hoc test between baseline and after 3 months. ^c^ Difference in a post hoc test between after RT and after 3 months. ^1^ In EORTC QLQ-C30: functional domains—higher scores are better; symptom domains—lower scores are better. ^2^ In EORTC BN20 symptom domains, lower scores are better.

**Table 3:** Correlations of the intensity of personality traits and anxiety as a trait with individual domains of quality of life at three time points of the study.

QoL Outcomes (*p*-Value; R Spearman)	Neuroticism			Extraversion			Psychoticism			Lie			STAI (Trade)		
	I	II	III	I	II	III	I	II	III	I	II	III	I	II	III
QLQ-C30 functional domains ^1^															
Global Health Status	0.012; −0.398	0.010; −0.412	0.163; −0.266	0.212; 0.204	0.615; 0.084	0.177; −0.258	0.663; 0.072	0.260; 0.187	0.255; −0.218	0.317; 0.164	0.453; 0.125	0.007; 0.491	<0.001; −0.650	<0.001; −0.656	0.074; −0.337
Physical Functioning	0.280; −0.177	0.559; −0.098	0.193; −0.249	0.662; 0.072	0.922; 0.016	0.017; −0.440	0.254; 0.187	0.105; 0.267	0.360; 0.176	0.900; −0.021	0.953; 0.010	0.300; 0.199	0.250; −0.189	0.151; −0.237	0.435; −0.151
Role Functioning	0.374; −0.146	0.306; −0.171	0.257; −0.217	0.535; 0.102	0.096; 0.274	0.986; 0.003	0.329; 0.160	0.698; 0.065	0.862; 0.034	0.547; 0.099	0.585; 0.091	0.253; 0.219	0.059; −0.305	0.083; −0.285	0.795; −0.051
Emotional Functioning	<0.001; −0.558	<0.001; −0.644	0.082; −0.328	0.194; 0.213	0.022; 0.369	0.241; −0.225	0.260; 0.185	0.276; 0.181	0.472; −0.139	0.879; 0.025	0.664; 0.073	0.028; 0.409	<0.001; −0.725	<0.001; −0.810	0.072; −0.339
Cognitive Functioning	0.138; −0.242	0.033; −0.346	0.158; −0.269	0.883; −0.024	0.291; 0.176	0.362; −0.176	0.937; −0.013	0.319; 0.166	0.930; 0.017	0.686; −0.067	0.558; −0.098	0.569; 0.110	0.001; −0.525	0.004; −0.455	0.093; −0.318
Social Functioning	0.002; −0.491	0.063; −0.304	0.291; −0.203	0.078; 0.286	0.244; 0.194	0.265; −0.214	0.993; 0.001	0.697; −0.065	0.969; 0.008	0.171; 0.224	0.380; 0.146	0.034; 0.396	0.001; −0.514	<0.001; −0.566	0.614; −0.098
QLQ-C30 symptom domains ^1^															
Fatigue	0.174; 0.222	0.363; 0.152	0.769; 0.057	0.129; −0.248	0.747; −0.054	0.054; 0.361	0.592; −0.089	0.172; −0.226	0.623; −0.095	0.060; −0.304	0.178; −0.223	0.072; −0.339	0.122; 0.252	0.024; 0.366	0.789; 0.052
Nausea and Vomiting	0.015; 0.387	0.043; 0.330	0.257; 0.217	0.091; −0.275	0.437; −0.130	0.986; −0.003	0.862; 0.029	0.271; −0.183	0.862; −0.034	0.436; −0.129	0.299; −0.173	0.253; −0.219	0.002; 0.476	0.255; 0.189	0.795; 0.051
Pain	0.273; 0.180	0.342; 0.159	0.578; 0.108	0.934; 0.014	0.599; 0.088	0.026; 0.413	0.210; −0.205	0.244; −0.194	0.199; −0.246	0.383; −0.144	0.235; −0.197	0.048; −0.370	0.410; 0.136	0.501; 0.113	0.998; 0.000
Dyspnea	0.098; 0.269	0.108; −0.323	0.816; 0.045	0.331; −0.160	0.048; −0.323	0.145; 0.277	0.610; 0.084	0.824; 0.037	0.862; −0.034	0.866; −0.028	0.078; −0.290	0.410; −0.159	0.036; 0.336	0.016; 0.389	0.993; 0.002
Insomnia	0.167; 0.226	0.011; 0.406	0.033; 0.396	0.155; −0.232	0.674; 0.070	0.693; 0.077	0.876; −0.026	0.438; −0.130	0.826; 0.043	0.894; 0.022	0.586; −0.091	0.020; −0.430	0.190; 0.215	0.003; 0.462	0.054; 0.361
Appetite Loss	0.030; 0.347	0.038; 0.338	0.086; 0.325	0.660; −0.073	0.285; 0.178	0.886; −0.028	0.925; −0.015	0.104; −0.268	0.743; −0.064	0.196; −0.212	0.794; −0.044	0.028; −0.409	0.087; 0.278	0.216; 0.205	0.447; 0.147
Constipation	0.304; 0.169	0.137; 0.246	0.421; 0.155	0.199; −0.210	0.756; −0.052	0.124; 0.292	0.726; −0.058	0.359; −0.153	0.855; −0.036	0.835; 0.035	0.664; −0.073	0.635; −0.092	0.186; 0.216	0.158; 0.233	0.693; 0.076
Diarrhea	0.106; −0.263	0.361; −0.152	0.768; 0.057	0.085; 0.279	0.090; 0.279	0.369; 0.173	0.590; 0.089	0.843; 0.033	0.765; −0.058	0.112; −0.259	0.356; −0.154	0.204; −0.243	0.194; −0.212	0.138; −0.245	0.167; 0.264
Financial Difficulties	0.901; −0.021	0.950; −0.010	0.731; −0.067	0.734; −0.056	0.701; −0.064	0.088; 0.322	0.621; −0.082	0.602; −0.087	0.551; −0.115	0.046; −0.322	0.049; −0.322	0.005; −0.506	0.621; −0.082	0.635; −0.080	0.106; −0.306
BN20 symptom domains ^2^															
Future Uncertainty	0.115; 0.276	0.152; 0.342	0.358; 0.246	0.118; −0.273	0.159; −0.336	0.192; 0.344	0.867; −0.030	0.997; −0.001	0.254; −0.303	0.627; −0.086	0.974; −0.008	0.080; −0.450	0.177; 0.237	0.060; 0.439	0.446; 0.205
Headaches	0.753; 0.056	0.014; 0.554	0.769; 0.080	0.447; 0.135	0.605; −0.127	0.249; 0.306	0.545; −0.107	0.331; −0.236	0.635; −0.128	0.242; −0.206	0.765; 0.074	0.020; −0.576	0.949; −0.011	0.036; 0.484	0.410; 0.221
Visual Disorders	0.605; −0.092	0.389; 0.210	0.763; 0.082	0.303; 0.182	0.016; 0.542	0.450; 0.203	0.892; −0.024	0.542; −0.149	0.595; −0.144	0.508; 0.117	1.000; 0.000	0.487; −0.188	0.908; −0.021	0.328; 0.237	0.361; 0.245
Seizures	0.360; 0.162	0.728; 0.085	0.879; 0.041	0.431; −0.140	0.152; −0.342	0.924; −0.026	0.681; −0.073	0.960; −0.012	0.682; 0.111	0.105; −0.283	0.805; 0.061	0.089; −0.439	0.118; 0.273	0.216; 0.298	0.160; 0.368
Motor Dysfunction	0.009; 0.442	0.413; 0.200	0.591; −0.145	0.712; −0.066	0.985; 0.005	0.851; 0.051	0.086; −0.299	0.034; −0.487	0.065; −0.473	0.377; 0.156	0.789; −0.066	0.584; −0.148	0.058; 0.329	0.257; 0.273	0.776; −0.077
Communication deficit	0.196; 0.227	0.892; −0.034	0.717; 0.098	0.915; −0.019	0.763; −0.074	0.806; −0.067	0.166; 0.243	0.369; 0.218	0.408; 0.222	0.918; −0.018	0.888; 0.035	0.648; −0.124	0.038; 0.357	0.608; 0.126	0.453; 0.202
Drowsiness	0.052; 0.336	0.843; 0.049	0.542; −0.165	0.387; −0.153	0.235; −0.286	0.140; 0.386	0.586; −0.097	0.200; −0.307	0.764; −0.082	0.972; 0.006	0.103; −0.385	0.074; −0.458	0.032; 0.368	0.191; 0.314	0.713; −0.100
Hair Loss	0.017; −0.407	0.440; −0.188	0.575; −0.152	0.437; 0.138	0.979; −0.006	0.040; 0.518	0.703; 0.068	0.903; 0.030	0.581; −0.149	0.372; −0.158	0.881; −0.037	0.303; −0.275	0.315; −0.178	0.972; −0.009	0.327; −0.262
Itchy Skin	0.682; −0.073	0.734; −0.083	0.464; −0.197	0.177; −0.237	0.806; −0.060	0.556; −0.159	0.338; −0.169	0.765; 0.073	0.893; −0.037	0.660; 0.078	0.557; −0.144	0.879; −0.042	0.264; 0.197	0.322; 0.240	0.119; −0.406
Weakness of Legs	0.142; 0.257	0.873; 0.039	0.073; −0.460	0.798; 0.045	0.584; 0.134	0.149; 0.378	0.164; −0.244	0.580; −0.135	0.588; −0.147	0.183; −0.234	0.053; −0.451	0.001; −0.734	0.198; 0.226	0.744; 0.080	0.260; −0.300
Bladder Control	0.371; 0.158	0.164; 0.332	0.818; −0.063	0.512; 0.117	0.794; 0.064	0.284; 0.285	0.174; −0.239	0.224; −0.292	0.219; −0.325	0.826; 0.039	0.846; −0.048	0.707; −0.102	0.981; −0.004	0.324; 0.239	0.973; 0.009

^1^ In EORTC QLQ-C30: functional domains—higher scores are better; symptom domains—lower scores are better. ^2^ In EORTC BN20 symptom domains, lower scores are better. I Assessment before RT. II Assessment one day after RT. III Assessment 3 months after RT.

**Table 4:** Multivariate linear regression models for quality of life measures.

Predictors (*p*-Value; Beta (ß))	Global Health Status			Physical Function			Role Function			Emotional Function			Cognitive Function			Social Function		
	I	II	III	I	II	III *	I	II	III *	I	II	III	I	II	III *	I	II	III *
Sex (woman)	0.846; 0.032	0.571; −0.076	0.494; −0.127	0.724; 0.074	0.960; −0.011	-	0.519; 0.134	0.885; −0.031	-	0.031; −0.322	0.564; −0.064	0.641; 0.104	0.455; −0.160	0.106; −0.304	-	0.792; −0.044	0.783; −0.056	-
Age	0.169; −0.200	0.394; −0.099	0.114; −0.298	0.162; −0.258	0.227; −0.225	-	0.997; −0.001	0.292; −0.197	-	0.001; −0.467	0.016; −0.241	0.082; −0.399	0.441; −0.141	0.104; −0.262	-	0.209; −0.181	0.544; −0.106	-
Education (secodary)	0.805; −0.040	0.619; −0.066	0.981; 0.004	0.065; −0.389	0.659; −0.093	-	0.825; −0.044	0.964; 0.010	-	0.138; 0.208	0.380; 0.096	0.623; −0.102	0.646; 0.095	0.228; 0.220	-	0.943; 0.011	0.202; −0.259	-
Education (vocational)	0.992; −0.001	0.087; 0.205	0.912; −0.016	0.343; 0.173	0.140; 0.279	-	0.518; 0.115	0.048; 0.385	-	0.018; 0.307	0.020; 0.235	0.803; 0.043	0.604; −0.095	0.110; 0.259	-	0.106; 0.236	0.157; 0.253	-
Education (high)	0.824; −0.030	0.834; 0.023	0.091; 0.239	0.674; −0.073	0.209; −0.226	-	0.269; −0.190	0.103; −0.299	-	0.170; −0.163	0.590; −0.049	0.974; 0.005	0.791; 0.046	0.665; 0.065	-	0.858; 0.024	0.837; 0.034	-
Tumor grade	0.020; −0.612	<0.001; −0.766	0.501; −0.179	0.019; −0.783	0.010; −0.882	-	0.001; −1.176	0.022; −0.782	-	0.997; −0.001	0.006; −0.488	0.863; −0.055	0.229; −0.391	0.052; −0.554	-	0.013; −0.660	0.188; −0.407	-
Chemotherapy (yes)	0.077; 0.402	0.027; 0.415	0.776; −0.066	0.034; 0.619	0.051; 0.575	-	0.009; 0.770	0.038; 0.620	-	0.700; −0.073	0.281; 0.159	0.659; −0.123	0.210; 0.360	0.502; 0.163	-	0.018; 0.547	0.434; 0.211	-
STAI trait	<0.001; −0.960	<0.001; −1.042	0.026; −0.531	0.087; −0.406	0.005; −0.725	-	0.001; −0.845	0.023; −0.569	-	<0.001; −0.731	<0.001; −0.894	0.212; −0.340	0.001; −0.856	0.002; −0.689	-	0.011; −0.492	0.002; −0.739	-
Neuroticism	0.454; 0.135	0.094; 0.250	0.072; −0.399	0.623; −0.112	0.162; 0.330	-	0.207; 0.286	0.270; 0.260	-	0.936; 0.012	0.737; 0.040	0.023; −0.628	0.343; 0.220	0.315; 0.200	-	0.328; −0.176	0.723; 0.078	-
Extraversion	0.339; 0.130	0.174; 0.152	0.049; −0.310	0.148; 0.253	0.082; 0.312	-	0.069; 0.315	0.006; 0.524	-	0.145; 0.172	0.001; 0.337	0.046; −0.380	0.829; 0.037	0.041; 0.317	-	0.005; 0.408	0.270; 0.184	-
Psychoticism	0.426; 0.120	0.348; 0.114	0.008; −0.483	0.827; −0.041	0.336; 0.187	-	0.371; 0.167	0.954; −0.011	-	0.516; −0.083	0.860; −0.017	0.032; −0.453	0.985; 0.004	0.805; −0.041	-	0.120; −0.236	0.379; −0.161	-
Lie	0.002; 0.458	<0.001; 0.473	<0.001; 0.725	0.060; 0.328	0.068; 0.323	-	0.064; 0.316	0.049; 0.354	-	0.006; 0.338	<0.001; 0.427	0.003; 0.631	0.975; 0.005	0.081; 0.263	-	0.001; 0.518	0.020; 0.396	-

* Regression model statistically insignificant. I Assessment before RT. II Assessment one day after RT. III Assessment 3 months after RT.

## Data Availability

The datasets generated for this study are available upon request to the corresponding author.

## References

[1] Noll K., King A.L., Dirven L., Armstrong T.S., Taphoorn M.J.B., Wefel J.S. (2022). Neurocognition and Health-Related Quality of Life Among Patients with Brain Tumors. Hematol. Oncol. Clin. N. Am..

[2] Louis D.N., Perry A., Reifenberger G., von Deimling A., Figarella-Branger D., Cavenee W.K., Ohgaki H., Wiestler O.D., Kleihues P., Ellison D.W. (2016). The 2016 World Health Organization Classification of Tumors of the Central Nervous System: A summary. Acta Neuropathol..

[3] Taphoorn M.J.B., Klein M. (2004). Cognitive deficits in adult patients with brain tumours. Lancet Neurol..

[4] Anbu Meena S., Srividya A., Kannan A., Krithika C.L., Aniyan Y. (2021). Assessment of quality of life in patients receiving radiotherapy: A multicentric study. J. Int. Oral Health.

[5] Antoniadis D., Giakoustidis A., Papadopoulos V., Fountoulakis K.N., Watson M. (2024). Quality of life, distress and psychological adjustment in patients with colon cancer. Eur. J. Oncol. Nurs..

[6] Langford D.J., Morgan S., Cooper B., Paul S., Kober K., Wright F., Hammer M.J., Conley Y.P., Levine J.D., Miaskowski C. (2020). Association of personality profiles with coping and adjustment to cancer among patients undergoing chemotherapy. Psychooncology.

[7] Iuso S., Monacis L., Nappi L., Malerba S., D’Andrea G., Altamura M., Margaglione M., Bellomo A., Petito A. (2022). Associations Between Personality Traits, Perceived Stress and Depressive Symptoms in Gynecological Cancer Patients Characterized by the Short and Long Allele Variant of the 5-HTTLPR Genotype: Preliminary Results. Clin. Neuropsychiatry.

[8] İzci F., Sarsanov D., Erdogan Z.İ., İlgün A.S., Çelebi E., Alço G., Kocaman N., Ordu Ç., Öztürk A., Duymaz T. (2018). Impact of Personality Traits, Anxiety, Depression and Hopelessness Levels on Quality of Life in the Patients with Breast Cancer. Eur. J. Breast Health.

[9] Honorato N.P., Abumusse L.V.d.M., Coqueiro D.P., Citero V.d.A. (2017). Personality Traits, Anger and Psychiatric Symptoms Related to Quality of Life in Patients with Newly Diagnosed Digestive System Cancer. Arq. Gastroenterol..

[10] de Mol M., Visser S., Aerts J., Lodder P., van Walree N., Belderbos H., den Oudsten B. (2020). The association of depressive symptoms, personality traits, and sociodemographic factors with health-related quality of life and quality of life in patients with advanced-stage lung cancer: An observational multi-center cohort study. BMC Cancer.

[11] Cerezo M.V., Blanca M.J., Ferragut M. (2020). Personality Profiles and Psychological Adjustment in Breast Cancer Patients. Int. J. Environ. Res. Public Health.

[12] de Pinho L.G., Lopes M.J., Correia T., Sampaio F., Arco H.R.d., Mendes A., Marques M.d.C., Fonseca C. (2021). Patient-Centered Care for Patients with Depression or Anxiety Disorder: An Integrative Review. J. Pers. Med..

[13] Knauer K., Bach A., Schäffeler N., Stengel A., Graf J. (2022). Personality Traits and Coping Strategies Relevant to Posttraumatic Growth in Patients with Cancer and Survivors: A Systematic Literature Review. Curr. Oncol..

[14] Gillis C., Ilie G., Mason R., Bailly G., Lawen J., Bowes D., Patil N., Wilke D., Rutledge R.D.H., Bell D. (2021). Personality Traits and Urinary Symptoms Are Associated with Mental Health Distress in Patients with a Diagnosis of Prostate Cancer. Curr. Oncol..

[15] Pichler T., Marten-Mittag B., Hermelink K., Telzerow E., Frank T., Ackermann U., Belka C., Combs S.E., Gratzke C., Gschwend J. (2022). Distress in hospitalized cancer patients: Associations with personality traits, clinical and psychosocial characteristics. Psychooncology.

[16] Wintraecken V.M., Vulik S., de Wild S., Dirksen C., Koppert L.B., de Vries J., Smidt M.L. (2022). A descriptive systematic review of the relationship between personality traits and quality of life of women with non-metastatic breast cancer. BMC Cancer.

[17] Wang H., Sun X., Yue H., Yang Y., Feng D. (2022). The dyadic effects of personality traits on depression in advanced lung cancer patients and caregivers: The mediating role of acceptance of illness. Eur. J. Cancer Care.

[18] Gempt J., Bette S., Albertshauser J., Cammardella J.H., Gradtke C., Wiestler B., Schirmer L., Ryang Y.-M., Meyer B., Ringel F. (2018). Personality Traits in Patients with Neuroepithelial Tumors—A Prospective Study. Sci. Rep..

[19] Bunevicius A. (2018). Personality traits, patient-centered health status and prognosis of brain tumor patients. J. Neurooncol..

[20] Louis D.N., Perry A., Wesseling P., Brat D.J., Cree I.A., Figarella-Branger D., Hawkins C., Ng H.K., Pfister S.M., Reifenberger G. (2021). The 2021 WHO Classification of Tumors of the Central Nervous System: A summary. Neuro Oncol..

[21] Scaringi C., Agolli L., Minniti G. (2018). Technical Advances in Radiation Therapy for Brain Tumors. Anticancer. Res..

[22] García-González J.M., Fernández-Muñoz J.J., Vergara-Moragues E., García-Moreno L.M. (2021). Eysenck Personality Questionnaire Revised-Abbreviated: Invariance gender in Spanish university students. EJREP.

[23] Spielberger C.D., Gorsuch R.L., Lushene R., Vagg P.R., Jacobs G.A. (1983). Manual for the State-Trait Anxiety Inventory.

[24] Aaronson N.K., Ahmedzai S., Bergman B., Bullinger M., Cull A., Duez N.J., Filiberti A., Flechtner H., Fleishman S.B., de Haes J.C. (1993). The European Organization for Research and Treatment of Cancer QLQ-C30: A quality-of-life instrument for use in international clinical trials in oncology. J. Natl. Cancer Inst..

[25] Osoba D., Aaronson N.K., Muller M., Sneeuw K., Hsu M.A., Yung W.K., Brada M., Newlands E. (1996). The development and psychometric validation of a brain cancer quality-of-life questionnaire for use in combination with general cancer-specific questionnaires. Qual. Life Res..

[26] Faul F., Erdfelder E., Lang A.-G., Buchner A. (2007). G*Power 3: A flexible statistical power analysis program for the social, behavioral, and biomedical sciences. Behav. Res. Methods.

[27] Rimmer B., Balla M., Dutton L., Williams S., Lewis J., Gallagher P., Finch T., Burns R., Araújo-Soares V., Menger F. (2024). “It changes everything”: Understanding how people experience the impact of living with a lower-grade glioma. Neurooncol. Pract..

[28] Lang-Rollin I., Berberich G. (2018). Psycho-oncology. Dialogues Clin. Neurosci..

[29] Malvaso A., Kang W. (2022). The relationship between areas of life satisfaction, personality, and overall life satisfaction: An integrated account. Front. Psychol..

[30] Kang W., Whelan E., Malvaso A. (2023). Understanding the Role of Cancer Diagnosis in the Associations between Personality and Life Satisfaction. Healthcare.

[31] Macía P., Gorbeña S., Gómez A., Barranco M., Iraurgi I. (2020). Role of neuroticism and extraversion in the emotional health of people with cancer. Heliyon.

[32] Chang H.-J., Chen W.-X., Lin E.C.-L., Tung Y.-Y., Fetzer S., Lin M.-F. (2014). Delay in seeking medical evaluations and predictors of self-efficacy among women with newly diagnosed breast cancer: A longitudinal study. Int. J. Nurs. Stud..

[33] Dahl A.A., Fosså S.D. (2022). High Neuroticism Is Related to More Overall Functional Problems and Lower Function Scores in Men Who Had Surgery for Non-Relapsing Prostate Cancer. Curr. Oncol..

[34] Rochefort C., Hoerger M., Turiano N.A., Duberstein P. (2019). Big Five personality and health in adults with and without cancer. J. Health Psychol..

[35] García-Torres F., Castillo-Mayén R. (2019). Differences in Eysenck’s Personality Dimensions between a Group of Breast Cancer Survivors and the General Population. Int. J. Environ. Res. Public. Health.

[36] Paulhus D.L. (2002). Socially desirable responding: The evolution of a construct. The Role of Constructs in Psychological and Educational Measurement.

[37] Grassi L. (2020). Psychiatric and psychosocial implications in cancer care: The agenda of psycho-oncology. Epidemiol. Psychiatr. Sci..

[38] Lengacher C.A., Reich R.R., Paterson C.L., Shelton M., Shivers S., Ramesar S., Pleasant M.L., Budhrani-Shani P., Groer M., Post-White J. (2019). A Large Randomized Trial: Effects of Mindfulness-Based Stress Reduction (MBSR) for Breast Cancer (BC) Survivors on Salivary Cortisol and IL-6. Biol. Res. Nurs..

[39] Chayadi E., Baes N., Kiropoulos L. (2022). The effects of mindfulness-based interventions on symptoms of depression, anxiety, and cancer-related fatigue in oncology patients: A systematic review and meta-analysis. PLoS ONE.

[40] Zhang L., Liu X., Tong F., Zou R., Peng W., Yang H., Liu F., Yang D., Huang X., Yi L. (2022). Cognitive behavioral therapy for anxiety and depression in cancer survivors: A meta-analysis. Sci. Rep..

[41] Hilfiker R., Meichtry A., Eicher M., Nilsson Balfe L., Knols R.H., Verra M.L., Taeymans J. (2018). Exercise and other non-pharmaceutical interventions for cancer-related fatigue in patients during or after cancer treatment: A systematic review incorporating an indirect-comparisons meta-analysis. Br. J. Sports Med..

[42] Dietrich J., Winter S.F., Parsons M.W. (2019). Delayed Neurologic Complications of Brain Tumor Therapy. Oncology of CNS Tumors.

[43] Soffietti R., Pellerino A., Bruno F., Mauro A., Rudà R. (2023). Neurotoxicity from Old and New Radiation Treatments for Brain Tumors. Int. J. Mol. Sci..

[44] Zwinkels H., Dirven L., Vissers T., Habets E.J.J., Vos M.J., Reijneveld J.C., van den Bent M.J., Taphoorn M.J.B. (2016). Prevalence of changes in personality and behavior in adult glioma patients: A systematic review. Neurooncol Pract..

